# Comparison of Bleaching Products With Up to 6% and With More Than 6% Hydrogen Peroxide: Whitening Efficacy Using BI and WI_*D*_ and Side Effects – An *in vitro* Study

**DOI:** 10.3389/fphys.2019.00919

**Published:** 2019-08-21

**Authors:** Bledar Lilaj, Rinet Dauti, Hermann Agis, Martina Schmid-Schwap, Alexander Franz, Fabian Kanz, Andreas Moritz, Andreas Schedle, Barbara Cvikl

**Affiliations:** ^1^Department of Conservative Dentistry and Periodontology, University Clinic of Dentistry, Medical University of Vienna, Vienna, Austria; ^2^Department of Prosthodontics, University Clinic of Dentistry, Medical University of Vienna, Vienna, Austria; ^3^Center for Forensic Medicine, Medical University of Vienna, Vienna, Austria; ^4^Department of Conservative Dentistry, Sigmund Freud University, Vienna, Austria

**Keywords:** bleached index, cytotoxicity, enamel surface morphology, hydrogen peroxide, SEM, tooth bleaching, whiteness index

## Abstract

The aim of this study was to evaluate the effect of bleaching agents containing different concentrations of hydrogen peroxide (HP) on color-change and on enamel-surface in bovine teeth. Furthermore the influence on cell viability and proliferation was investigated. Two hundred and forty teeth were randomly assigned into four groups (home bleaching ≤6%, in-office bleaching ≤6%, in-office bleaching > 6% HP, and control group). Bleaching was performed after artificial staining and the bleached index (BI) as well as the whiteness index (WI_*D*_) was measured at several time points. Chemical analysis for HP concentrations and the pH of the bleaching products was done. Furthermore, enamel surfaces of randomly selected specimens were analyzed using scanning electron microscopy (SEM) and cytotoxicity of the tested bleaching products was evaluated *in vitro* using dental pulp cells (DPCs) and L929 cells. A statistically significant whitening effect was observed in almost all products. As expected all investigated products resulted in decreased cell viability, however, with different values of LC50 (median lethal concentration). SEM analysis showed an analog of enamel alterations with decreasing pH, increasing exposure time, and increasing HP concentration. Bleaching agents containing a low HP concentration are considered to be effective and to have less damaging effects on enamel and tested cells.

## Introduction

Tooth bleaching has grown considerably in importance in the daily routine of general dentistry due to the increased esthetic awareness of patients. Since various factors can cause tooth discoloration different treatment alternatives may be required. Extrinsic tooth stains caused by consumption of coloring consumables such as black tea, red wine, and cigarettes can be removed by professional oral hygiene. On the other hand, intrinsic tooth discoloration resulting e.g., from the disintegration of hemoglobin after a dental trauma or medications like Tetracyclines (Botelho et al., 2017) require chemical auxiliaries to break large color pigments (Nathoo, 1997). The most commonly used chemical agents for tooth bleaching are hydrogen peroxide (HP) and carbamide peroxide. The resulting bleaching effect depends on the peroxide concentration, the application mode, and the exposure time (Matis et al., 2006; Meireles et al., 2012).

Hydrogen peroxide directly reacts to color pigments, while carbamide peroxide releases one third of its volume in form of HP, thus constituting the active bleaching agent (Dahl and Pallesen, 2003; Goldberg et al., 2010). Bleaching is achieved by splitting large chromogenic pigments by active oxygen radicals gained from the HP (Seghi and Denry, 1992). The resulting small molecules reflect more light, which makes the tooth appear brighter (Joiner, 2006; Ozkan et al., 2013). The active HP concentration plays a crucial role for the resulting bleaching effect, but it can also have possible side effects. Thus, several legal directives and recommendations for concentrations of HP used in dentistry have been established (Siew, 2000; Union TCotE, 2011). For safety reasons, higher concentrations (more than 0.1% HP) require the application by the dentist and can be provided to the patient for the rest of the bleaching cycle. Agents containing low concentrations can be directly purchased by patients.

Therefore, the application mode depends on the concentration of HP. In-office bleaching products usually contain HP concentrations about 30–40%, and should therefore be exclusively applied and supervised by the professional (Kwon and Wertz, 2015). The use of a light source for its application is optional, since it does not seem to improve and accelerate the whitening effect (Maran et al., 2017), but it may allow stable whitening effects in combination with low concentrated bleaching products (Vildosola et al., 2017a). Home bleaching products mostly contain a maximum concentration of 6% HP. In order to avoid side effects on soft and hard tissue, a clinical examination has to be carried out prior to every bleaching treatment. In-office products are directly applied on the enamel surface after protecting the soft tissue with a rubber dam. Home bleaching products are usually applied on custom-made splints which are filled with the bleaching agent and then placed on the teeth (Carey, 2014).

Hence, there are two methods of vital tooth bleaching, in-office and home-bleaching that can also be combined together (Rezende et al., 2016). Depending on the final HP concentration, the exposure time varies between several minutes up to numerous days in total. Most in-office bleaching products are applied up to 1 h, including the replacement of the bleaching agent in 2–3 cycles. Home-bleaching products, on the other hand, are applied for up to 8 h for multiple days. Higher concentrated bleaching products achieve the brightening effect in shorter time; however, they are associated with a higher risk of possible side effects.

Dental hypersensitivity after bleaching is painful and therefore one of the most uncomfortable side effects (Ray, 2014). Increasing HP concentrations show higher risk of tooth sensitivity (Lima et al., 2018). Nevertheless, it is reversible within a few days (Vildosola et al., 2017b) and literature demonstrates that the addition of different agents may reduce these without compromising the whitening effect (Kossatz et al., 2012). Another critical side effect that needs to be taken into consideration when performing bleaching is the cytotoxicity of bleaching agents. Depending on the composition and the HP percentage within different bleaching products, toxicity has been observed in different cell linages. Several studies have investigated cytotoxic effects of HP on dental pulp cells (DPCs) (Dantas et al., 2010; Lima et al., 2013; Duque et al., 2014) and fibroblasts (Dantas et al., 2010; Duque et al., 2014; Baldea et al., 2017) reporting variable results in cell viability (Duque et al., 2014). Negative effects on hard tissue are manifested as alteration of the enamel surface resulting in rough microstructure and demineralization (Ogura et al., 2013; Luque-Martinez et al., 2016) requiring different methods of remineralization (Kemaloglu et al., 2014). Depending on the extent of the alterations, they may cause increased adherence of bacteria and extrinsic staining molecules.

For an optimal bleaching result without any side effects, an ideal interplay of peroxide concentration, application mode, and exposure time is a sine qua non. The aim of the present study was the exploration of the whitening effect, of the effects on the enamel surface, as well as the cytotoxicity of different bleaching products, containing from 0% up to 40% HP *in vitro*.

## Materials and Methods

### Preparation of the Samples

**FIGURE 1:**
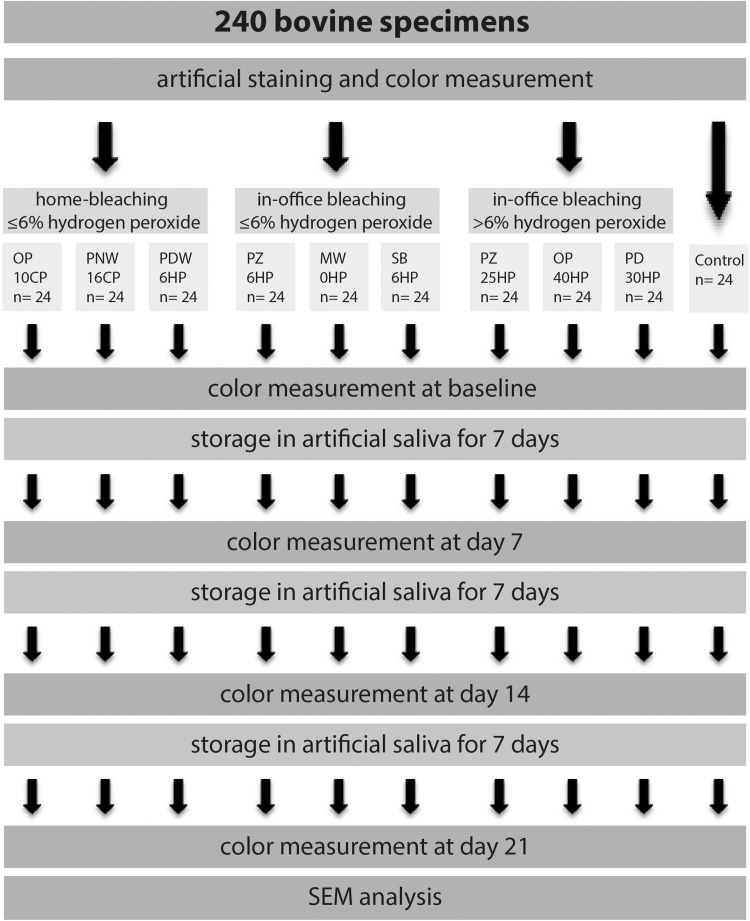
Illustration of the experimental design, starting with artificial staining of a total of 240 bovine specimens. Teeth were divided into three experimental groups, including three bleaching products each, and one control group. Tooth color was determined immediately after completed bleaching procedure, as well as 7, 14, and 21 days after.

The experimental procedure of the present study is illustrated in Figure 1. Two hundred and forty extracted bovine incisors were selected for this study. Only sound teeth without cracks were used. The roots were sectioned 2 mm apically to the cementoenamel junction and then stored overnight under refrigeration (4°C) in a saturated thymol solution until preparation for testing. The entire crowns were used after undergoing an artificial staining process. A mixture of 1l of red wine (Merlot IGT, Domenica, Veneto, Italy) and 1l black tea (English Breakfast, Teekanne GmbH & Co., KG, Düsseldorf, Germany) was prepared. The crowns were immersed into the staining solution for 7.5 days at 37°C (Kielbassa et al., 2009). Subsequently, all specimens were rinsed with water and stored in artificial saliva (NRF 7.5) (Apothekerkammer Sachsen-Anhalt, 1997) at 4°C for 1 week. After fixing the specimens on slides using a cold polymerizing resin (Technovit^®^ 4000, Heraeus Kulzer, Hanau, Germany), the buccal enamel surfaces were flat ground using a grinder (EXAKT, 400 CS) with 320-grit, and 1200-grit abrasive papers (Struers, Ballerup, Denmark) based on Borges et al. (2011). Polishing was performed using a polishing machine (MetaServ^®^ Grinder-Polisher, Buehler, Lake Bluff, IL, United States) with a 1.0 μm aluminia suspension (MicroPolish^TM^ II, Buehler, Lake Bluff, IL, United States), simulating oral hygiene before bleaching procedure. The resulting exposed enamel area was at least 6 mm in diameter. Previous to the bleaching procedure, specimens were cleaned using an ultrasonic cleaner (Tevion GT-7810A, Essen, Germany) for 90 s.

### Bleaching Procedures

A total of 216 specimens were randomly assigned to 3 groups containing three different bleaching products (*n* = 24). The first group included home-bleaching products containing less than or equal to 6% HP, the second group included in-office bleaching products containing less than or equal to 6% HP, and the third group included in-office bleaching products containing more than 6% HP. Each bleaching procedure was performed according to the manufacturer’s instructions. Details about the bleaching products are given in Tables 1A–C. Between the bleaching procedures specimens were stored in artificial saliva at 4°C. Furthermore, one additional group (*n* = 24) without any bleaching procedure acted as control. Tooth color was assessed both by means of the bleached index (BI), which is generated by a spectrophotometer, as well as the whiteness index (WI_*D*_) (Perez Mdel et al., 2016).

**TABLE 1A:** Information of the tested home-bleaching products containing ≤6% hydrogen peroxide (HP) according to the manufacturer regarding their HP concentration, pH value, and exposure time.

**Product name**	**Abbreviation**	**Company**	**Percentage HP**	**pH**	**Exposure time**
Opalescence^®^ PF 10%	OP 10CP	Ultradent Products, Inc., South Jordan, UT, United States	∼3.6%	6.5	6 h/day during 14 days
Philips ZOOM^®^! NiteWhite^®^ 16%	PNW 16CP	Philips, Amsterdam, Netherlands	∼5.8%	6.0–7.0	6 h/day for 7 days
Philips ZOOM^®^! DayWhite^®^ 6%	PDW 6HP	Philips, Amsterdam, Netherlands	6%	6.0–7.0	Twice a day for 30 min for a period of 14 days

**TABLE 1B:** Information of the tested in-office bleaching products containing ≤6% HP according to the manufacturer regarding their HP concentration, pH value, and exposure time.

**Product name**	**Abbreviation**	**Company**	**Percentage HP**	**pH**	**Exposure time**
Philips ZOOM^®^! 6%	PZ 6HP	Philips, Amsterdam, Netherlands	6% activated by a blue LED light (Philips Zoom^®^ WhiteSpeed)	7.0–8.5	Four times for 15 min
Magic White^®^ 0%	MW 0HP	Drogenbos, Belgium	0%	–	Cleaning specimens with a finger brush; prefabricated tray, inserted in the mouth and activated by a LED light for 21 min
Smartbleach^®^ 6%	SB 6HP	Smartbleach International, Herzele, Belgium	6% activated by a green LED light (Smartlight 3LT)	9.5–10.0	Activated three times for 9 min

**TABLE 1C:** Information of the tested in-office bleaching products containing >6% HP according to the manufacturer regarding their HP concentration, pH value, and exposure time.

**Product name**	**Abbreviation**	**Company**	**Percentage HP**	**pH**	**Exposure time**
Philips ZOOM^®^! 25%	PZ 25HP	Philips, Amsterdam, Netherlands	25% activated by a blue LED light (Philips Zoom^®^ WhiteSpeed)	7.0–9.0	Four times for 15 min
Opalescence^®^ Boost 40%	OP 40HP	Ultradent Products, Inc., South Jordan, UT, United States	40%	6.0–8.5	Three times for 20 min
Philips Dash 30%	PD 30HP	Philips, Amsterdam, Netherlands	30%	4.8–5.2	45 min

### Evaluation of the Color Change

The effectiveness of the different bleaching products was measured using the VITA Easyshade Advance 4.0 spectrophotometer (Vita Zahnfabrik, Bad Säckingen, Germany) utilizing the CIEL^∗^a^∗^b^∗^ system (Borges et al., 2015) before bleaching procedure, at baseline as well as 7, 14, and 21 days after bleaching, respectively. Baseline is defined as the time immediately after the completion of the whole bleaching cycle. The “L value” indicates the lightness of the color (0 = black, 100 = white), the “a value” indicates the color between green and red (−a = green, +a = red), and the “b value” indicates the color between blue and yellow (−b = blue, +b = yellow) (CIE CIDLE, 2004). For reproducibility purposes the tip of the spectrophotometer was placed in the center of every prepared enamel surface.

Two different bleached indices were used to disclose the results. The “bleached index,” which is automatically generated by the VITA Easyshade Advance 4.0, allows for easy control of a bleaching process. As recommended by the American Dental Association (ADA), to reveal the number of shade guide units achieved, the resulting difference is calculated by subtracting the BI measured after treatment from the BI before treatment. The second index used in the present study is the “Whiteness index,” which was especially developed for dentistry to correlate with tooth color perception. WI_*D*_ has outperformed previous whiteness indices in validation experiments. It is calculated using the following formula (Perez Mdel et al., 2016):

$$W\,I_{D}=0.511\,L^{*}-2.324\,a^{*}-1.100\,b^{*}$$

### Chemical Analysis of the Bleaching Products

Chemical analysis was conducted in order to confirm the manufacturer’s information regarding the concentration of the activated bleaching products. Therefore cerimetric titration, utilizing ferroin until color change, was performed for the determination of the HP content (Kwon et al., 2013). pH was analyzed using pH-Indicator Strips (MColorpHast^TM^, VWR International, PA, United States).

### SEM Analysis of Bleached Samples

Two specimens of each group were randomly selected and the surface was examined using a scanning electron microscope (FEI Quanta 200 FEGSEM, Hillsboro, Oregon, United States). Imaging of the polished enamel surface was performed at 3.000× and 10.000× magnification. The structure of the enamel surface was evaluated regarding morphology and signs of damage.

### Cell Viability and Cell Proliferation Testing

Testing for cell viability was performed using DPCs from three different donors and from L929 cells, a mouse fibroblast cell line. Having obtained informed consent (Ethical Committee, Medical University of Vienna, Vienna, Austria), human DPCs were isolated from caries-free teeth after wisdom tooth extraction in healthy individuals. Cells were cultured in Dulbecco’s modified Eagle’s minimum essential medium (DMEM; Invitrogen, Carlsbad, CA, United States) including 10% fetal bovine serum (FBS; Invitrogen), and antibiotics (Invitrogen) at 37°C; 5% CO_2_. For the viability testing cells were seeded at a density of 30,000 cells/cm^2^ on microtiter plates (Greiner Bio-One, Frickenhausen, Germany). 24 h later, bleaching gels that resulted in an effective whitening effect on the enamel samples in the experiments on color change were added to the cells. The concentrations of all bleaching products used are defined as % weight per volume. Cells were incubated with the tested bleaching gels in a dilution series (25–0.8% w/v) for 2 min (Table 2). Afterwards, the cells were rinsed with Phosphate-buffered saline (PBS) and suspended in serum-free medium. For viability testing, MTT [3-(4,5-dim- ethythiazol-2-yl)-2,5-diphenyltetrazolium bromide; Sigma- Aldrich St Louis, MO, United States] was added and cells were incubated for 2 h at 37°C. Due to a conversion of MTT into formazan crystals by the cells, viability was detectable by color changes after the christals were dissolved in dimethyl sulfoxide (DMSO). This change of color was measured (EL 808; Biotek Instruments, Winooski, VT, United States) and normalized to that of untreated cells. The half lethal concentration LC50 for every single bleaching gel was now calculated by an exponential regression analysis (Cvikl et al., 2015, 2017).

**TABLE 2:** Concentrations of HP used for cell experiments.

**% dilution**	**OP 10CP = 3.6HP**	**PNW 16CP = 5.8HP**	**PDW 6HP**	**PZ 6HP**	**PZ 25HP**	**OP 40HP**	**PD 30HP**
25.00	0.90	1.45	1.50	1.50	6.25	10.00	7.50
12.50	0.45	0.73	0.75	0.75	3.13	5.00	3.75
6.25	0.23	0.36	0.38	0.38	1.56	2.50	1.88
3.13	0.11	0.18	0.19	0.19	0.78	1.25	0.94
1.56	0.06	0.09	0.09	0.09	0.39	0.63	0.47
0.78	0.03	0.05	0.05	0.05	0.20	0.31	0.23

*Manufacturers indicate weight percent for OP 10CP and OP 40HP and percent for PNW 16CP, PDW 6HP, PZ 6HP, PZ 25HP, and PD 30HP.*

Additional evidence of cell survival or death was obtained after 1% w/v incubation with the corresponding bleaching gels. The cells were morphologically visualized, and images of the DPCs and L929 cells were taken directly after MTT addition. Cell death was evaluated using the trypan blue exclusion assay. Furthermore, a Live-Dead staining was performed using the Live-Dead Cell Staining Kit (Enzo Life Sciences AG, Lausen, TX, United States) according to the instructions of the manufacturer. A cell permeable green fluorescent dye stained live cells, while dead cells were stained by propidium iodide, a red fluorescent dye, which in viable cells is actively pumped out of the cytoplasm. Visualization of the Live-Dead staining was performed using fluorescence microscopy. Untreated cells served as negative control and the cells incubated with a 6% H_2_O_2_ solution served as positive control in all samples.

Testing the proliferation behavior of DPCs and L929 cells after incubation with the respective bleaching gels was performed in duplicates using a 5-Bromo-20-deoxyuridine incorporation assay. Bleaching gels at 1% w/v were added to the cells as described above. During incubation of the cells with 5-bromo-20-deoxyuridine (BrdU, Roche, Basel, Switzerland), BrdU was incorporated by proliferating cells and caused a color change. This change of color was measured (EL 808; Biotek Instruments, Winooski, VT, United States) and normalized to that of untreated cells.

### Statistical Analysis

The color (represented by the BI and the WI_*D*_) was detected using a spectrophotometer (VITA Easyshade Advance 4.0) utilizing the CIEL^∗^a^∗^b^∗^ system, before bleaching procedure, at baseline, as well as 7, 14, and 21 days after bleaching, respectively. Differences in color between the bleaching agents 21 days after bleaching procedure were analyzed using the non-parametric Mann-Whitney *U* test with Bonferroni correction for multiple comparisons. Differences in color before and 21 days after bleaching procedure were analyzed using the Wilcoxon-Signed Rank test. Results of the cell viability testing were used to calculate the half lethal concentration (LC50). LC50 values of different bleaching products were statistically analyzed using the Mann-Whitney *U* test with Bonferroni correction. The software SPSS 21.0 (SPSS Inc., Chicago, Illinois, United States) was used for statistical analysis. Descriptive analysis of the SEM samples was performed.

## Results

### Evaluation of the Color Change

**FIGURE 2:**
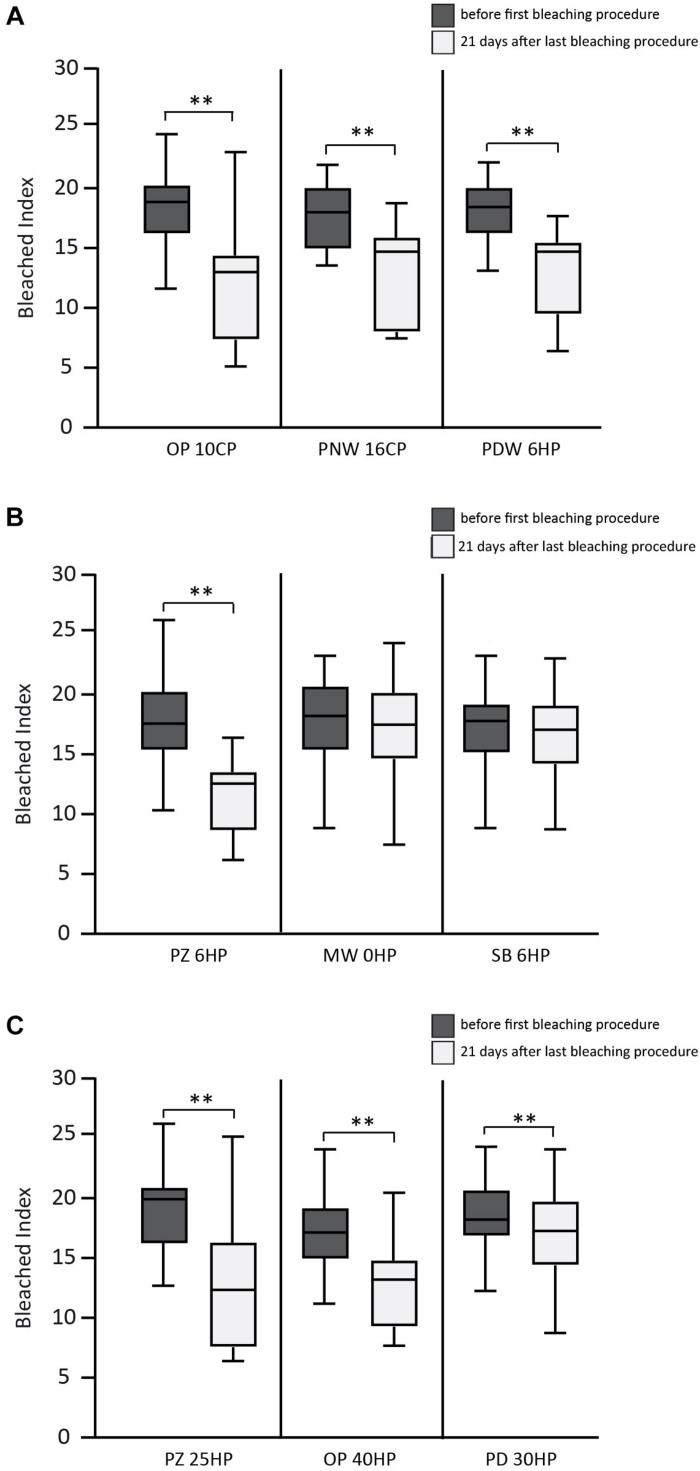
Results of the bleached index (BI) before and 21 days after bleaching procedure. **(A)** Overview of the results of the used home-bleaching products containing up to 6% hydrogen peroxide (HP) (Opalescence^®^ PF 10%, OP 10CP; Philips ZOOM^®^! NiteWhite^®^ 16%, PNW 16CP; and Philips ZOOM^®^! DayWhite^®^ 6%, PDW 6HP). **(B)** Results of in-office bleaching products containing up to 6% HP (Philips ZOOM^®^! 6%, PZ 6HP; Magic White^®^ 0%, MW 0HP and Smartbleach^®^ 6%, SB 6HP). **(C)** Results of in-office bleaching products containing more than 6% HP (Philips ZOOM^®^! 25%, PZ 25HP; Opalescence^®^ Boost 40%, OP 40HP and Philips Dash 30%, PD 30HP). Statistical significance highlighted by ^∗∗^*p* < 0.001.

The results of the BI before and 21 days after bleaching procedure are shown in Figure 2. After staining procedure and before bleaching no statistical significant difference in color was measured between all specimens. Regarding the control group (no bleaching procedure), no color changes were detected during the experimental time (data not shown).

In the first group (home-bleaching products containing up to 6% HP) the three tested products (Opalescence^®^ PF 10%, OP 10CP; Philips ZOOM®! NiteWhite^®^ 16%, PNW 16CP; and Philips ZOOM®! DayWhite^®^ 6%, PDW 6HP) showed a statistically significant whitening effect indicated by a decrease of the BI 21 days after bleaching (*p* < 0.001 each). No statistically significant differences between the three tested bleaching products were detected after 21 days.

In the second group (in-office bleaching products containing up to 6% HP) Philips ZOOM^®^! 6% (PZ 6HP) showed a statistically significant decrease of BI 21 days after bleaching (*p* < 0.001), while the other two products of this group showed no statistical significant difference. PZ 6HP resulted in lower BI compared to Magic White^®^ 0% (MW 0HP) and Smartbleach^®^ 6% (SB 6HP) (*p* < 0.001 each).

In the third group (in-office bleaching products containing more than 6% HP) Philips ZOOM^®^! 25% (PZ 25HP), Opalescence^®^ Boost 40% (OP 40HP), and Philips Dash 30% (PD 30HP) showed a statistically significant decrease of BI 21 days after bleaching (*p* < 0.001 each). Statistically significant differences were found between PZ 25HP and PD 30HP (*p* = 0.003) and between OP 40HP and PD 30HP (*p* = 0.001).

No statistical differences were detected in the comparison between the products with the highest decrease of BI in each group (OP 10CP, PZ 6HP, and PZ 25HP).

**FIGURE 3:**
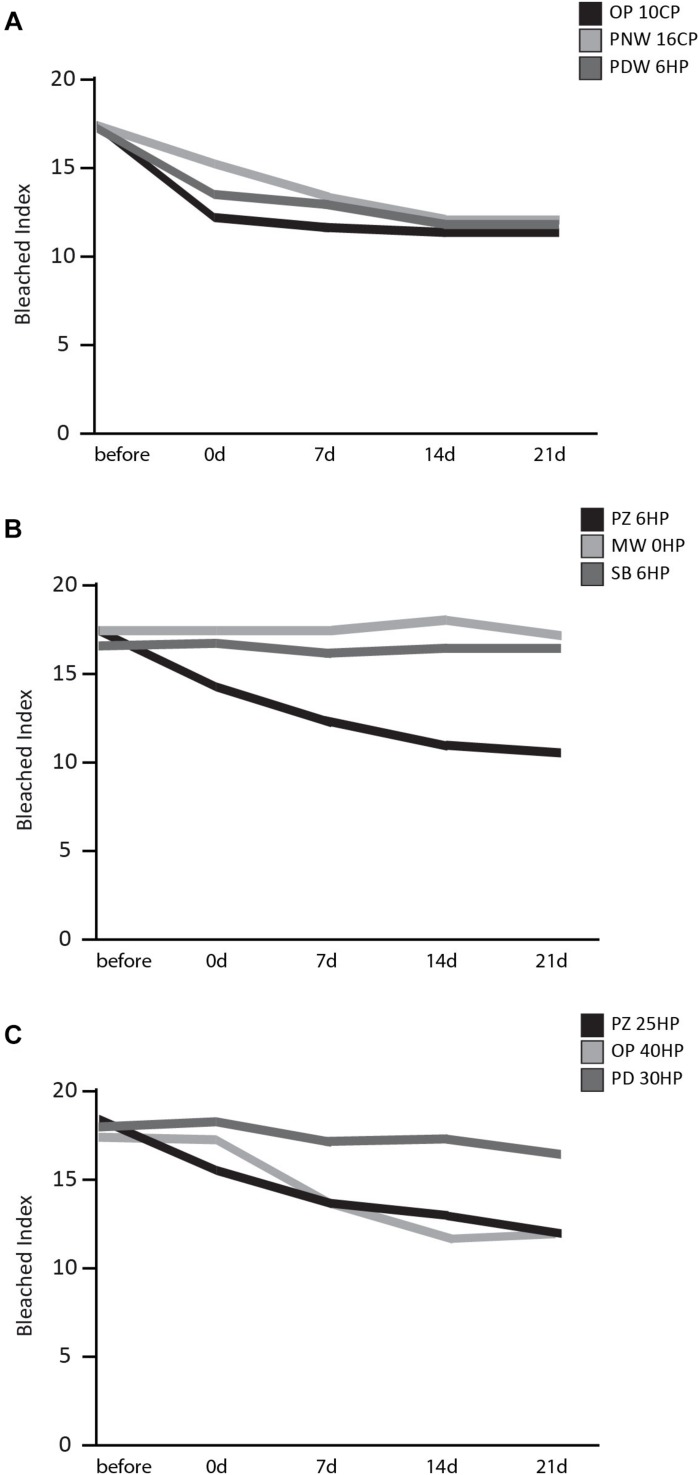
Results of the BI over a time period of 21 days after bleaching procedure. **(A)** Results of home-bleaching products containing up to 6% HP (Opalescence^®^ PF 10%, OP 10CP; Philips ZOOM^®^! NiteWhite^®^ 16%, PNW 16CP; and Philips ZOOM^®^! DayWhite^®^ 6%, PDW 6HP). **(B)** Results of in-office bleaching products containing up to 6% HP (Philips ZOOM^®^! 6%, PZ 6HP; Magic White^®^ 0%, MW 0HP and Smartbleach^®^ 6%, SB 6HP. **(C)** Presenting the results of the used in-office bleaching products containing more than 6% HP (Philips ZOOM®! 25%, PZ 25HP; Opalescence^®^ Boost 40%, OP 40HP and Philips Dash 30%, PD 30HP).

The bleached index over time of all three groups is illustrated in Figure 3. What is worth noting in this graph is that in group one all products (OP 10CP, PNW 16CP, and PDW 6HP) showed a decrease of BI directly after bleaching procedure. No obvious difference in bleaching effect occurred over the following 21 days (Figure 3A). In group two a reduction of BI directly after and also 7 days after bleaching was observed when using PZ 6HP. MW 0HP, and SB 6HP showed no difference over the whole period (Figure 3B). In group three (Figure 3C), PZ 25HP resulted in a decrease of BI directly after the bleaching procedure, whereas OP 40 produced a single bleaching effect after 7 days. PD 30HP showed a slight bleaching effect over the whole period.

Regarding WI_*D*_, all tested products were analog to the results of the BI.

In the first group all three products showed a statistically significant decrease of WI_*D*_ 21 days after bleaching (OP 10CP, *p* = 0.001; PNW 16CP and PDW 6HP, *p* < 0.001 each). Within this group, no statistically significant differences between the tested bleaching products were detected after 21 days. In the second group PZ 6HP showed a statistically significant decrease of WI_*D*_ 21 days after bleaching (*p* < 0.001). PZ 6HP resulted in a lower WI_*D*_ compared to MW 0HP and SB 6HP (*p* = 0.001 each). In the third group PZ 25HP (*p* < 0.001), OP 40HP (*p* < 0.001) and PD 30HP (*p* = 0.002) showed a statistically significant decrease of WI_*D*_ 21 days after bleaching. Statistically significant differences were detected between PZ 25HP and PD 30HP (*p* = 0.012) and between OP 40HP and PD 30HP (*p* < 0.001).

### Chemical Analysis of the Bleaching Products

The results of the measurement of the HP concentration are shown in Table 3.

**TABLE 3:** Hydrogen peroxide concentrations of all tested bleaching products as given by the manufacturer and measured by the authors.

	**HP in %, according to manufacturer**	**HP in %, measured**
**Home-bleaching ≤6% HP**		
OP 10CP	3.62	3.10
PNW 16CP	5.79	4.51
PDW 6HP	6.00	5.06
**In-office-bleaching ≤6% HP**		
PZ 6HP	6.00	4.53
MW 0HP	0.00	0.50
SB 6HP	6.00	7.80
**In-office-bleaching >6% HP**		
PZ 25HP	25.00	21.29
OP 40HP	40.00	40.72
PD 30HP	30.00	41.00

In the analysis of OP 10CP the HP content resulted in 3.10%, PNW 16CP in 4.51%, PDW 6HP in 5.06%, PZ 6HP in 4.53%, and PZ 25HP in 21.29%. These concentrations were equal to or less than those described in the manufacturer’s description. On the contrary, MW 0HP, SB 6HP, OP 40HP, and PD 30HP showed higher HP concentration levels than those described in the manufacturer’s description; 0.50, 7.80, 40.72, and 41.00%, respectively.

The results of the performed pH-measurement are shown in Table 4.

**TABLE 4:** pH values of all tested bleaching products as given by the manufacturer and measured by the authors.

	**pH according to manufacturer**	**pH measured with MColorpHast**^TM^****
**Home-bleaching ≤6% HP**		
OP 10CP	6.5	6.5
PNW 16CP	6.0–7.0	5.0
PDW 6HP	7.0–8.5	8.5
**In-office-bleaching ≤6% HP**		
PZ 6HP	7.0–8.5	8.5
MW 0HP	–	5.0
SB 6HP	9.5–10	8.0
**In-office-bleaching >6% HP**		
PZ 25HP	7.0–9.0	9.0
OP 40HP	6.0–8.5	7.5
PD 30HP	4.8–5.2	5.0

PDW 6HP resulted in a pH of 8.5; PZ 6HP in 8.5; PZ 25HP in 9.0 OP 40HP in 7.5, and PD 30HP in 5.0. These results correspond with the data of the manufacturer’s description. The measured pH of OP 10CP resulted in a pH of 6.5, PNW 16CP 5.0, and SB 6HP 8.0, respectively. These findings showed lower pH than announced by the manufacturers. MW 0HP showed a pH of 5.0 while no manufacturer’s information was available.

### SEM Analysis of Bleached Samples

**FIGURE 4:**
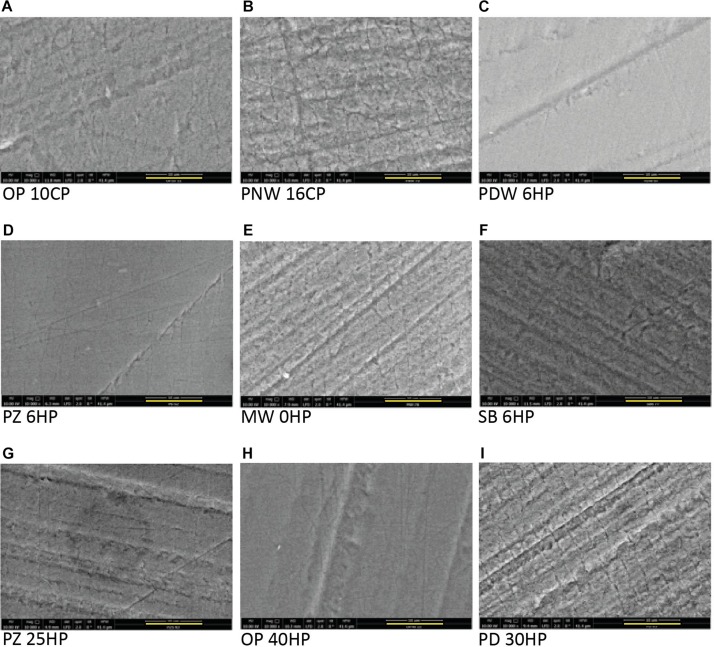
Scanning electron microscope micrographs of all tested products after the bleaching procedure showing a base roughness of all specimens due to the grinding procedure. All micrographs are illustrated in 10 000 fold magnification (the yellow bar represents 10 μm). **(A)** Opalescence^®^ PF 10%, OP 10CP. **(B)** Philips ZOOM^®^! NiteWhite^®^ 16%, PNW 16CP. **(C)** Philips ZOOM^®^! DayWhite^®^ 6%, PDW 6HP. **(D)** Philips ZOOM^®^! 6%, PZ 6HP. **(E)** Magic White^®^ 0%, MW 0HP. **(F)** Smartbleach^®^ 6%, SB 6HP. **(G)** Philips ZOOM^®^! 25%, PZ 25HP. **(H)** Opalescence^®^ Boost 40%, OP 40HP. **(I)** Philips Dash 30%, PD 30HP.

In the control group (no bleaching procedure), no morphological changes were detected during the experimental time. All micrographs presenting enamel surfaces after the bleaching procedures in Figure 4 are illustrated in 10 000 fold magnification (the bar represents 10 μm).

#### Home-Bleaching Products Containing ≤6% Hydrogen Peroxide

Surface morphology of enamel samples after bleaching with PNW 16CP (Figure 4B) showed erosive enamel alterations with increased micro roughness, while the bleaching with OP 10CP resulted in moderate alterations (Figure 4A). When PDW 6HP was used for bleaching treatment only slight morphological alterations were detected (Figure 4C).

#### In-Office Bleaching Containing ≤6% Hydrogen Peroxide

After bleaching enamel samples with MW 0HP accentuated alterations of the surface occurred, even resulting in visible enamel prisms (Figure 4E). When SB 6HP was applied for bleaching, moderate depositions on the surface appeared (Figure 4F). PZ 6HP resulted in no morphological enamel damage after the bleaching procedure (Figure 4D).

#### In-Office Bleaching Containing >6% Hydrogen Peroxide

The investigated enamel surface is characterized by slight morphological irregularities when OP 40HP was used as bleaching agent (Figure 4H). PZ 25HP resulted in moderate and accentuated alterations of the sample (Figure 4G), while severe erosive alterations were detected when PD 30HP was applied on the enamel surface (Figure 4I).

### Cell Viability and Cell Proliferation Testing

**FIGURE 5:**
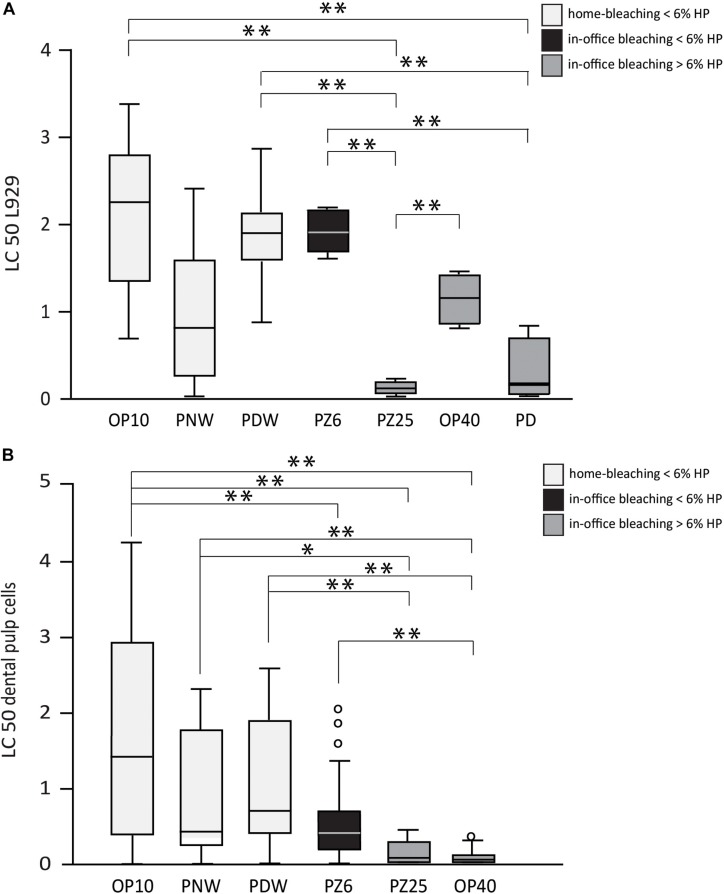
Half lethal concentration (LC 50) values of all tested bleaching products (Opalescence^®^ PF 10%, Philips ZOOM^®^! NiteWhite^®^ 16%, Philips ZOOM^®^! DayWhite^®^ 6%, Philips ZOOM^®^! 6%, Philips ZOOM^®^! 25%, Opalescence^®^ Boost 40%, Philips Dash 30%) under incubation of L929 cells **(A)** and dental pulp cells **(B)**. Statistical significance is highlighted by ^∗^*p* < 0.05 and ^∗∗^*p* < 0.001.

**FIGURE 6:**
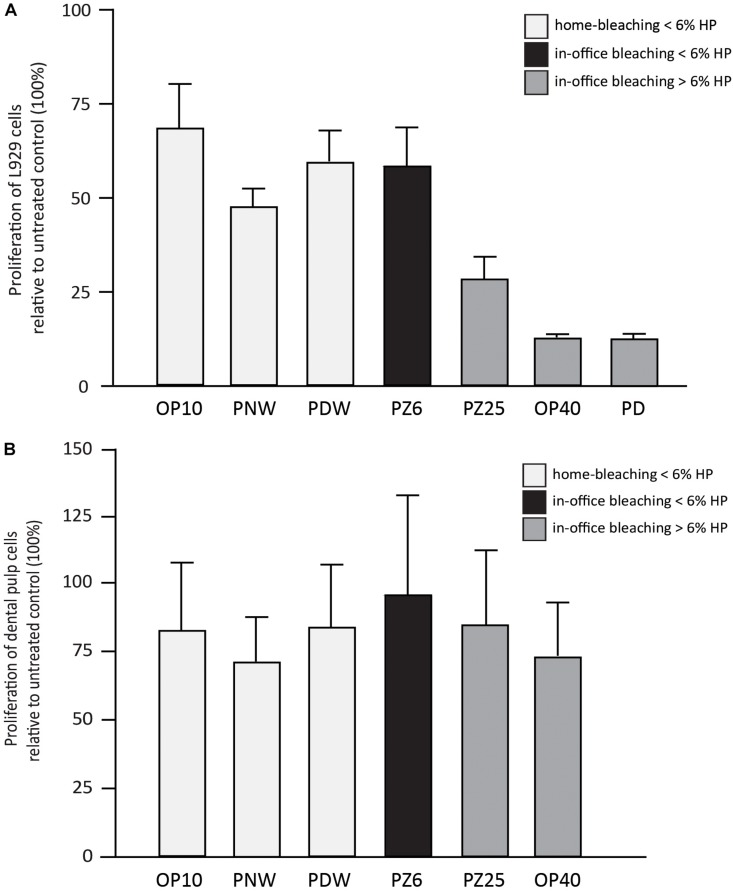
Results (in %) of L929 cell proliferation **(A)** and dental pulp cell proliferation **(B)** in comparison with the untreated control after incubation with 1% w/v of Opalescence^®^ PF 10%, Philips ZOOM^®^! NiteWhite^®^ 16%, Philips ZOOM^®^! DayWhite^®^ 6%, Philips ZOOM^®^! 6%, Philips ZOOM^®^! 25%, Opalescence^®^ Boost 40% and Philips Dash 30%.

The results of the cytotoxicity tests and the proliferation capacity of cells after incubation with the bleaching products are shown in Figures 5, 6. Only bleaching products were tested that achieved significant bleaching effects on the bovine enamel. In the performed cytotoxicity tests OP 10CP, PDW 6HP, and PZ 6HP resulted in statistical significant higher LC50 values in L929 cell line compared to PZ 25HP (all *p* < 0.001) and PD 30HP (*p* = 0.001, *p* < 0.001, and *p* < 0.001, respectively), whereas OP 40HP could demonstrate a significant higher LC50 value in comparison with PZ 25HP (*p* = 0.004) (Figure 5A). Investigating DPCs, all bleaching gels resulted in mean LC50 values below 2%. OP 10CP showed the highest mean LC50 values (1.6 ± 1.4) with significant higher values compared to PZ 6HP (*p* = 0.001), PZ 25HP (*p* < 0.001), and OP 40HP (*p* < 0.001). PZ 25HP and OP 40HP showed the lowest mean LC50 values (0.12 ± 0.1, 0.08 ± 0.1, respectively). PZ 25HP resulted in statistical significant lower LC50 values compared to OP 10CP (as described above), PDW (*p* < 0.001), and PNW (*p* = 0.003). OP 40HP resulted in statistical significant lower LC50 values compared to all other bleaching products (all *p* < 0.001), with exception of PZ 25HP (Figure 5B).

Regarding the proliferation behavior of the incubated cells, a descriptive analysis was performed, considering the smaller sample size (Figures 6A,B). The cell proliferation of L929 cells after incubation with the bleaching gels showed lower values when bleaching products with higher HP concentrations were used (Figure 6A). DPCs also resulted in less proliferation after incubation with the respective bleaching gels. However, DPCs seemed to be less sensitive (Figure 6B).

**FIGURE 7:**
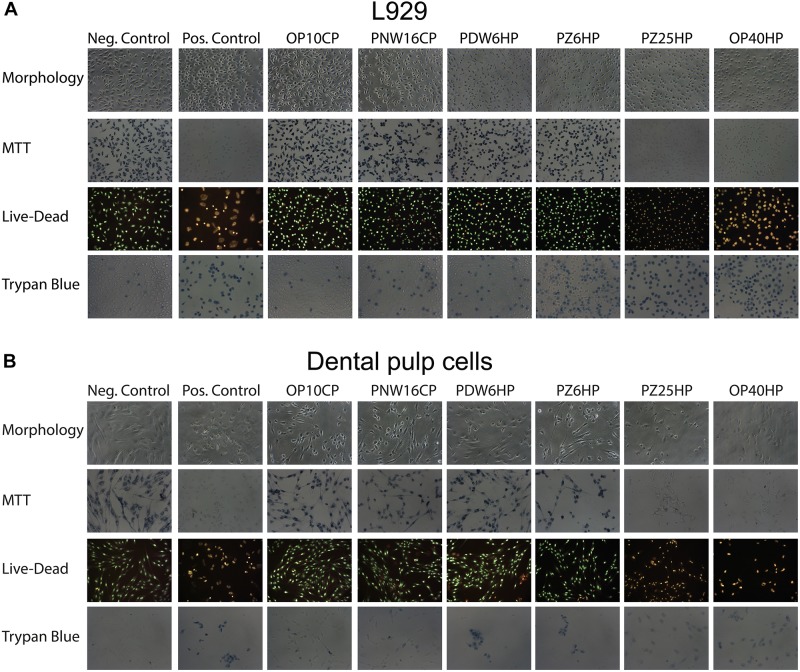
Cell morphology and the micrographs of L929 cells **(A)** and dental pulp cells **(B)** after adding MTT, performing Live-Dead staining and adding Trypan blue after incubation of the cells with 1%w/v of Opalescence^®^ PF 10%, Philips ZOOM^®^! NiteWhite^®^ 16%, Philips ZOOM^®^! DayWhite^®^ 6%, Philips ZOOM^®^! 6%, Philips ZOOM^®^! 25%, and Opalescence^®^ Boost 40%. Untreated cells and cells after adding 6% H_2_O_2_ served as negative and positive control, respectively.

Looking at the viability based on cell morphology, MTT staining, Live-Dead staining, and Trypan Blue Dye Exclusion Assay, the L929 and DPCs showed similar results; an association of lower cell viability with higher HP concentrations could be detected (Figures 7A,B).

## Discussion

Patient requests of tooth discolorations for esthetic reasons have become an increasing issue for dental practitioners in recent years (Luque-Martinez et al., 2016). Therefore, different variations of tooth whitening products are available on the market. The bleaching agent as well as its concentration, its application mode and the associated exposure time may influence the effectiveness of the bleaching products, and cause side effects on the tooth surface (Ogura et al., 2013; Cvikl et al., 2016). Hence, the aim of the present study was to investigate the effect of nine bleaching products with different peroxide concentrations, application modes and exposure times and furthermore, to investigate the exposed enamel surface regarding morphological irregularities.

The nine bleaching products tested were divided into three groups according to their HP concentration and their application mode. In the group of home-bleaching products containing less than or equal to 6% HP, no statistic significant differences between the tested bleaching products (OP 10CP, PNW 16CP, and PDW 6HP) were detected. These results are partially in accord with a study by Carlos et al. (2017) and Aka and Celik, 2017, which also showed an effective whitening effect when using Opalescence^®^ PF 10% (OP 10CP). In contrast, in the in-office group containing less than or equal to 6% HP, Philips ZOOM^®^! 6% (PZ 6HP) showed significantly better whitening effects compared to Magic White^®^ 0% (MW 0HP), and Smartbleach^®^ 6% (SB 6HP). A possible explanation for these different bleaching results might be the different shapes of the light sources as well as HP concentrations ranging from 0 to 6%. Philips ZOOM^®^! 25% (PZ 25HP), Opalescence^®^ Boost 40% (OP 40HP), and Philips Dash 30% (PD 30HP), all three products of the in-office group containing more than 6% HP, resulted in significant tooth whitening. These results are in agreement with those obtained by Park et al. (2016), who achieved solid whitening effects using PZ 25 with light activation.

Of particular clinical interest is the finding that the most effective bleaching products (OP 10CP, PZ 6HP, and PZ 25HP) of the three groups (home-bleaching ≤6%, in-office bleaching ≤6%, and in-office bleaching >6%) solely revealed no statistically significant differences. An explanation might be that lower concentrations of HP, due to the longer exposure time, resulted in similar whitening effects like the products with higher concentration. High concentrated HP whitening products have been well investigated regarding enamel damage (Uthappa et al., 2012; Coceska et al., 2016), also have different remineralization methods (Kemaloglu et al., 2014; Coceska et al., 2016). However, in the present study most notably the pH, ranging from 5.0 to 8.5, and the exposure time seem to influence enamel surface morphology. In the groups containing in-office bleaching agents, ≤6% and >6%, where exposure time was comparable, pH seems to be a determining factor affecting enamel irregularities. Therefore, a neutral pH is recommended in bleaching materials, since acidic agents can lead to irregularities of the enamel morphology (Pimenta-Dutra et al., 2017). In literature it is also discussed whether the enhancement of the mechanical properties of enamel is affected by the addition of different modifiers into the bleaching gels (Borges et al., 2009; Borges et al., 2011; Santos et al., 2016) or by the use of remineralizing agents (Coceska et al., 2016). However, this was not evaluated in the present study. It may be argued that the use of bovine teeth instead of human teeth may have limited the findings of our study. However, according to the systematic review and meta-analysis by Soares et al. (2016), bovine teeth can suitably substitute human teeth with regard to their enamel, and dentin properties.

Besides alterations in hard tissue, cell cytotoxicity of bleaching products remains a critical issue, since contact with soft tissue cannot always be excluded, especially when using home bleaching trays. Different extent of cytotoxicity caused by HP has been described by several studies (Dantas et al., 2010; Lima et al., 2013; Duque et al., 2014). As expected, the results of this study demonstrate a tendency of higher HP concentrations causing more cytotoxic effects, which was also confirmed by de Almeida et al. (2015). Regarding the viability and proliferation tests using DPCs and L929 cells, both reveal a rather decreased toxic effect of products containing ≤6% HP compared to those agents containing >6% HP. These results are in accordance with findings in other studies in which the tendency of higher concentrated products to be more cytotoxic is also reported (Llena et al., 2018). At any rate, the LC50 values calculated on the basis of the viability testing even showed a toxic potential of highly diluted bleaching solutions ranging from 1 to 2%. Therefore, the overall difference in effect may not be over interpreted. In the proliferation testing assays, L929 cells supported the overall finding that the concentration of the used HP strongly influences the behavior of the cells. Interestingly, the proliferative capacities of DPCs seem to be more resistant against the bleaching gels at 1% w/v. A possible explanation for this controversy could be a kind of “struggle for survival” reaction of the cells that might occur more often in DPCs than in fibroblasts. Nevertheless, the results of this study suggest in-office products to be safer, since in-office bleaching allows for a more controlled application of bleaching gel. Besides, the exposure time is minimized.

The control samples showed no change in tooth color during the whole experimental period, proving that no additional whitening effect occurred due to a potential washout procedure by the storage in artificial saliva. Regarding the results, in the home-bleaching group (≤6% HP) all three agents achieved a whitening effect directly after the bleaching procedure. In the in-office group ≤6% HP the use of PZ 6HP resulted in a color change at two points in time, immediately after bleaching, and 7 days after. MW 0HP, SB 6HP had no effect at any time. In the in-office group >6% HP, P25 HP also showed an effect directly after the bleaching procedure, while OB 40HP presented an increase in whitening 7 days after completion of bleaching. PD 30HP showed a slight bleaching effect over the whole period. These results support the recommendation of a delayed color determination. Especially in case of planned subsequent, esthetic composite restorations, the clinician should allow for treatment in extended periods of time. A waiting period of at least 2 weeks as recommended by Wilson et al. (2009) or 3 weeks as recommended by Cavalli et al. (2001) after the completed bleaching procedure will also ensure adequate bond strength (Bijelic-Donova et al., 2015). Contrary to several studies (Gegauff et al., 1993; Rosenstiel et al., 1996), no color rebound appeared after a hydration period of 21 days, which is however, in accordance with the findings of a clinical study by Ontiveros et al. (2012) showing no color relapse at all.

It is worth noting that the present study revealed analog results when using BI and WI_*D*_ for investigating the effect of the bleaching procedures. While BI is a suitable index for communicating the achieved whitening effect to the patient, WI_*D*_ is appropriate for the measurement, and evaluation of whiteness for scientific purposes (Perez Mdel et al., 2016). This may be an important issue for future research, since both, the clinical as well as the scientific color change determination are covered with these two indices.

## Conclusion

The results of the present study show that products with low HP concentration, neutral to alkaline pH, and combined with a short exposure time can be recommended for tooth whitening. Furthermore, studies are necessary to enforce the use of BI as a patient-friendly index and of WI_*D*_ as a scientific index.

## Data Availability

The datasets generated for this study are available on request to the corresponding author.

## Ethics Statement

We used an established protocol for cell isolation. Informed and written consent was given by the donors. The protocol was approved by the Ethics Committee of the Medical University of Vienna (631/2007). Regarding the bovine teeth an ethical review process was not required for this study, because teeth were obtained from an abattoir. No animal was sacrificed for this study.

## Author Contributions

BL, BC, and AS designed the study. BL, BC, HA, and FK collected the data. BL, BC, RD, and HA analyzed and interpreted the data. MS-S, AF, FK, and AM contributed to the discussion of the manuscript. BL, BC, and RD drafted the manuscript. All authors revised the manuscript. BC and AS supervised the study.

## Conflict of Interest Statement

The authors declare that the research was conducted in the absence of any commercial or financial relationships that could be construed as a potential conflict of interest.
